# The scaffold-dependent function of RIPK1 in experimental non-alcoholic steatohepatitis

**DOI:** 10.1007/s00109-022-02217-z

**Published:** 2022-06-15

**Authors:** Valeria Pistorio, Juliette Tokgozoglu, Vlad Ratziu, Jérémie Gautheron

**Affiliations:** 1 Department of Chemical Sciences, University of Naples Federico II, Naples, Italy; 2 Centre de Recherche Saint-Antoine (CRSA), Sorbonne Université, Inserm Paris, France; 3 Institute of Cardiometabolism and Nutrition (ICAN), Paris, France; 4 Department of Hepatology, Pitié-Salpêtrière Hospital, Assistance Publique-Hôpitaux de Paris (AP-HP), Paris, France; 5 Centre de Recherche Des Cordeliers (CRC), Sorbonne Université, Inserm Paris, France

**Fig. 1:**
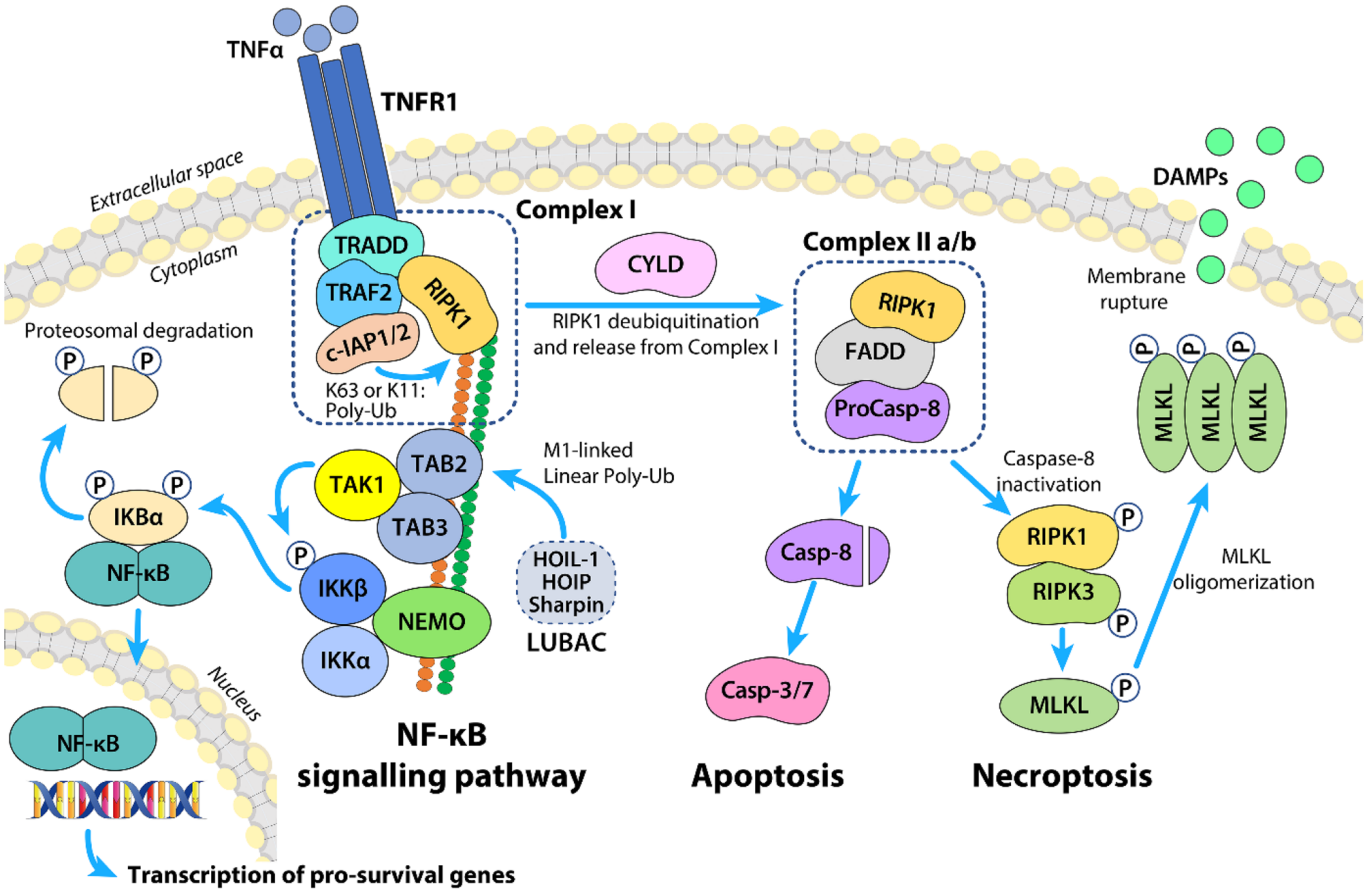
The role of RIPK1 in TNF signaling pathway. RIPK1 is a key upstream regulator at the crossroads of inflammation and cell death regulating NF-κB signaling pathway, apoptosis, and necroptosis. The recognition of TNF-α by TNFR1 receptor triggers the rapid assembly of a multiprotein complex known as complex I, which consists of adaptor proteins (e.g., TRADD, FADD, TAB), ubiquitin ligases (e.g., TRAF2, LUBAC, cIAPs), and kinases (e.g., TAK and RIPK1) [4]. In complex I, RIPK1 is ubiquitylated by cIAP1/2 (K63 or K11) [5] and LUBAC (M1 linked linear Poly-Ub), thereby serving as an enzymatically inactive scaffold for the recruitment of TAK1 and NEMO, the regulatory subunit of the IKK complex. TAK1 is essential for IKKβ phosphorylation that leads to the phosphorylation, ubiquitylation, and proteasomal degradation of the inhibitory protein IKBα, thereby allowing NF-κB to translocate to the nucleus and drive the transcription of pro-survival genes. When the cellular environment favors the transition from a state of inflammation to death, the deubiquitylation of RIPK1 by CYLD, and its release from complex I promotes the formation of complex IIa (5), which ultimately results in caspase-8-dependent apoptosis. When caspases or cIAPs are inhibited, RIPK1 associates with RIPK3 to form the necrosome (complex IIb), which in turn recruits the pseudokinase MLKL. RIPK3-mediated phosphorylation of MLKL results in MLKL translocation to the plasma membrane and pore formation, causing membrane permeabilization and ultimately leading to necroptosis. cIAPs, cellular inhibitor of apoptosis proteins; CYLD, cylindromatosis; FADD, Fas associated death domain protein; IKK, IκB kinase; LUBAC, linear ubiquitin chain assembly complex; MLKL, mixed lineage kinase domain like pseudokinase; NEMO, NF-kappa-B essential modulator; RIPK, receptor-interacting serine/threonine-protein kinase; TAB, TAK1-binding protein; TAK1, transforming growth factor β-activated protein kinase 1; TRADD, tumor necrosis factor receptor type 1-associated DEATH domain protein; TRAF2, tumor necrosis factor receptor-associated factor 2

Non-alcoholic fatty liver disease (NAFLD) is the most common chronic liver disease with increasing prevalence worldwide [1]. NAFLD is defined by hepatic fat accumulation in greater than 5% of hepatocytes. In a subset of NAFLD patients, non-alcoholic steatohepatitis (NASH) can develop, a condition characterized by hepatocellular injury and liver inflammation. Long-standing steatohepatitis leads to persistent inflammation and fibrosis, which eventually leads to cirrhosis and hepatocellular carcinoma (HCC) [1]. Despite the significant burden on the global public health system, no FDA-approved drugs are currently available for NASH, and liver transplantation remains the ultimate therapeutic option [1]. The presence of hepatocyte cell death, reflected by increased levels of serum alanine- and aspartate-aminotransferase, is a cardinal feature of NASH [2]. Until recently, two main forms of cell death were recognized: apoptosis, which occurs in a highly controlled manner, and necrosis, which is accidentally triggered. However, in recent years, it has become clear that programmed cell death was not restricted to apoptosis, but comprised other forms of regulated cell death [3]. Necroptosis is one of them, combining the molecular machinery of the extrinsic apoptotic pathways with an execution similar to necrosis (Fig. 1). Unlike apoptosis that requires the activation of aspartate-specific proteases known as caspases, necroptosis is first driven by the activation of the receptor-interacting protein kinase (RIPK) 1 and 3, followed by the activation of the pseudo kinase mixed lineage kinase domain-like (MLKL) [3]. RIPK1 has emerged as a key upstream regulator at the crossroads of inflammation and cell death regulating NF-κB signaling pathway, apoptosis, and necroptosis (Fig. 1) [4, 5]. The ability of RIPK1 to modulate these key cellular events is tightly controlled by post-translational modifications, and RIPK1 acts both as a scaffolding protein and a kinase [4, 5]. In mice, genetic ablation of *Ripk1* results in early postnatal lethality with widespread cell death in lymphoid and adipose lineages [6] while RIPK1 kinase-dead knock-in mice survive to adulthood without impairment and being resistant to necroptosis [5]. This duplicity has been evocated in disease contexts where cell-specific depletion vs. inhibition of RIPK1 kinase activity raise interesting questions. In the liver, the functions of RIPK1 have remained vague until now.

In the paper from the group of Michel Samson published in the present issue of *Journal of Molecular Medicine* [7], the authors examined the role of RIPK1 in NASH by applying a conditional cre/loxP-based knockout approach of RIPK1 in parenchymal liver cells. The authors have previously described this conditional knock-out model, confirming that *Ripk1*^LPC−KO^ mice are viable and reach adulthood without morphological alterations of the liver [8]. These findings emphasize that RIPK1 in liver parenchymal cells is not essential for maintaining liver homeostasis under physiological conditions. Nevertheless, *Ripk1*^LPC−KO^ mice developed fulminant hepatitis associated with increased hepatocyte apoptosis when low doses of Concanavalin A or TNF-α were administrated [8]. This phenotype could be reverted with the administration of a pan-caspase inhibitor [8]. Similarly, *Ripk1*^LPC−KO^ mice developed more severe symptoms at an early stage of viral-induced fulminant hepatitis, and administration of poly I:C (a viral-like immunostimulant of toll-like receptor-3) triggered increased of transaminases in *Ripk1*^LPC−KO^ mice, reflecting liver damage through induced apoptosis [9]. The neutralization of TNF-α as well as macrophage depletion were able to prevent hepatocyte apoptosis in poly I:C-challenged *Ripk1*^LPC−KO^ mice [9]. These investigations support the pro-survival scaffolding function of RIPK1 in hepatocytes, at least in acute liver injury, by preventing excessive activation of apoptosis.

In the current study, Farooq et al. fed WT and *Ripk1*^LPC−KO^ mice a high-fat-high-cholesterol (HFHC) diet. This feeding recapitulates key histological changes seen in human NASH, including hepatocyte ballooning and injury, hepatic inflammation, and liver fibrosis [10]. The authors found that HFHC diet was associated with an upregulation of RIPK1, of death ligands, and their receptors in WT mice, confirming the relevance of this dietary model to study the cell death functions of RIPK1. They did not, however, find differences in body weight gain, liver injury, and steatosis between WT and *Ripk1*^LPC−KO^ mice when fed a HFHC diet. In addition, the inflammatory response and the liver immune cell populations were unchanged. Surprisingly, there was a significant increase in liver fibrosis in *Ripk1*^LPC−KO^ mice after 12 weeks of HFHC diet, shown both by Sirius red staining and the expression of TGF-β, a key inducer of hepatic stellate cells (HSCs) trans-differentiation into scar-forming myofibroblasts. Of note, increased fibrosis was not due to an increase in collagen 1 deposition in the KO mice, but rather to a significant overexpression of Timp1 and Timp2, two tissue inhibitors of metalloproteinases, involved in degradation of the extracellular matrix, suggesting an altered balance between matrix synthesis and degradation in *Ripk1*^LPC−KO^ mice. These findings highlight that inflammation does not always coexist with fibrosis and suggest alternative pathways of myofibroblast activation. In this case, cell products specifically released by knock-out cells might influence the epithelial/stromal interactions leading to an over-production of matrix. NF-κB signaling pathways, where RIPK1 plays a critical role as a scaffolding protein, could be a privileged target to study this interaction between hepatocytes and HSCs.

Given the assumption that hepatocyte death is the key trigger of liver disease progression towards fibrosis, and the privileged role of RIPK1 in regulating cell death, it is surprising that *Ripk1* ablation in liver parenchymal cells did not reduce apoptosis. Although the level of apoptosis did not differ between WT and *Ripk1*^LPC−KO^ mice, other types of regulated cell death such as necroptosis could have been induced in *Ripk1*^LPC−KO^ mice and contribute to worsening fibrosis, a hypothesis not tested in the present work. Alternatively, cholangiocytes, which are more sensitive than hepatocytes to necrosis, could participate to the phenotype through the release of specific cell products upon their death, which could then activate myofibroblasts [11, 12]. It would also be interesting to evaluate the level of hepatic cell death induced by a HFHC diet. Further studies are therefore needed to decipher the cell death pathways triggered in the absence of RIPK1 in fatty livers.

The metabolic effects of the HFHC diet and the potential impact of *Ripk1* ablation are worth investigating. Indeed, whole-body *Mlkl* deficiency has been shown to prevent obesity-induced insulin resistance and glucose intolerance in mice fed a high fat diet (HFD) [13]. In addition, chemical inhibition of RIPK1 or its knockdown using an oligonucleotide antisense (ASO) improved hepatic insulin sensitivity as well as insulin resistance and glucose intolerance in leptin-deficient (*ob/ob*) and HFD-fed mice [13, 14]. Therefore, there is evidence to suggest that RIPK1 may have cell-death-independent effects in influencing whole-body and hepatic-specific metabolism.

From this point of view, there are, however, divergent results from other studies. For instance, WT mice fed a HFD and treated with an oligonucleotide antisense (ASO) to knock down RIPK1 showed a substantial reduction of key inflammatory genes downstream of RIPK1 [14]. Also, therapeutic silencing of RIPK1 using ASO dramatically reduced fat mass in HFD fed mice [14]. The discrepancy between the latter study and the results of Farooq et al. could be explained by the divergent functions that RIPK1 might have in different cell types. In chronic liver diseases, non-parenchymal cells (NPC), namely HSC-myofibroblasts and macrophages, participate in the progression of fibrosis. In this regard, Tao et al. demonstrated that RIPK1 was highly expressed in liver macrophages of human NASH and that its kinase activity was critical to the involvement of hematopoietic-derived macrophages in the pathology of murine steatohepatitis [15]. Moreover, chemical inhibition of RIPK1 kinase activity significantly alleviated steatohepatitis [16], acute liver injury [17], and acute on chronic liver failure (ACLF) [18]). Also, ubiquitinated RIPK1 can form a platform which facilitates the activation of multiprotein complexes, such as NF-κB and MAPK. This platform appears important for the integrity of hepatocyte function as RIPK1 knock-out sensitizes hepatocytes to TNF-mediated apoptosis [8]. It is therefore not surprising that in many animal models of liver diseases, genetic or pharmacological inhibition of RIPK1 had different effects than RIPK1 ablation specifically in liver parenchymal cells. In the latter case, compensatory mechanisms to the absence of RIPK1 could have resulted in fibrosis worsening.

The evidence presented by Farooq et al. contributes to a better understanding of the role of RIPK1 scaffolding function in the development of NASH. It clearly confirmed the duplicity of RIPK1 which is both a scaffolding protein and a kinase, with the kinase function already known to contribute to the pathogenesis of NASH. Nevertheless, further studies are needed to evaluate the differential contribution of RIPK1 in NPC vs. parenchymal cells and whether pharmacological inhibition can hold promise as a therapy for NASH.
